# Comparison between binocular indirect ophthalmoscopy and digital retinography for diabetic retinopathy screening: the multicenter Brazilian Type 1 Diabetes Study

**DOI:** 10.1186/s13098-015-0110-8

**Published:** 2015-12-21

**Authors:** Fernando Korn Malerbi, Paulo Henrique Morales, Michel Eid Farah, Karla Rezende Guerra Drummond, Tessa Cerqueira Lemos Mattos, André Araújo Pinheiro, Felipe Mallmann, Ricardo Vessoni Perez, Franz Schubert Lopes Leal, Marília Brito Gomes, Sergio Atala Dib, On behalf of The Brazilian Type 1 Diabetes Study Group

**Affiliations:** Departments of Endocrinology and Ophthalmology, Federal University of São Paulo, São Paulo, Brazil; Department of Ophthalmology, Federal University of São Paulo, São Paulo, Brazil; Department of Ophthalmology, Hospital Federal dos Servidores do Estado, Rio de Janeiro, Brazil; Department of Ophthalmology, Centro de Endocrinologia e Diabetes do Estado da Bahia, Salvador, Brazil; Department of Ophthalmology, Hospital Regional de Taguatinga, Brasília, Brazil; Department of Ophthalmology, Federal University of Rio Grande do Sul, Porto Alegre, Brazil; Department of Endocrinology, University of São Paulo, São Paulo, Brazil; Department of Ophthalmology, University of Campinas, Campinas, Brazil; Department of Endocrinology, State University of Rio de Janeiro, Rio de Janeiro, Brazil; Department of Endocrinology, Federal University of São Paulo, São Paulo, Brazil

**Keywords:** Diabetic retinopathy, Telemedicine, Screening, Retinography, Blindness

## Abstract

**Background:**

Diabetic retinopathy is the main cause of preventable blindness in the economically active population in western countries. Diabetic retinopathy screening is effective in preventing blindness and can be performed through various diagnostic methods. Our objective is to compare binocular indirect ophthalmoscopy (BIO) to telemedicine protocols of digital retinography for diabetic retinopathy screening in a large and heterogenous type 1 diabetes population in a developing country.

**Methods:**

Data from 1266 Type 1 Diabetes Mellitus patients from a Brazilian multicenter study were analyzed. Patients underwent BIO and digital retinography, non-mydriatic and mydriatic. Images were sent to a reading center in a telemedicine protocol. Agreement between the different methods was calculated with kappa statistic for diabetic retinopathy and maculopathy classification. Clinical outcome was either observation or referral to specialist.

**Results:**

Agreement between BIO and mydriatic retinography was substantial (kappa 0.67–0.74) for diabetic retinopathy observation vs referral classification. Agreement was fair to moderate (kappa 0.24–0.45) between retinography and BIO for maculopathy. Poor mydriasis was the main obstacle to image reading and classification, especially on the non-mydriatic strategy, occurring in 11.9 % of right eyes and 16.9 % of left eyes.

**Conclusion:**

Mydriatic retinography showed a substantial agreement to BIO for diabetic retinopathy observation vs referral classification. A significant amount of information was lost on the non-mydriatic technique because of poor mydriasis. We recommend a telemedicine-based diabetic retinopathy screening strategy with digital mydriatic retinography, preferably with 2 fields, and advise against non-mydriatic retinography in developing countries.

## Background

Diabetic retinopathy (DR) is one of the main causes of preventable blindness in the world, affecting 12.6 million and threatening the vision of 37.3 million in 2010 [1]. It is the main cause of preventable blindness in the economically active population in developed countries [2]. Diabetes mellitus (DM) accounts for more than 10 % of the health care budget of some countries, with DR being one of the main expenditures related to DM.

DR screening has been considered to have an excellent cost-effectiveness [3, 4], allowing the detection of early stages of the disease, preventing visual impairment and decreasing the economic burden of DR treatment [5–9].

DR may impair vision in its advanced stages and also because of diabetic macular edema, which may occur at any stage of this diabetic chronic complication. DR can be diagnosed by binocular indirect ophthalmoscopy (BIO), with or without slit-lamp biomicroscopy, or through retinal photographs (retinographies) [10]. The Early Treatment Diabetic Retinopathy Study (ETDRS) established a gold standard of 7-field stereoscopic photographs for the diagnosis of DR [11].

Brazil, a country of continental dimensions, has approximately 12 million people between 20 and 79 years old with diabetes [12]. There are currently no nation-wide studies of DR prevalence in Brazil, but available data point to a prevalence ranging from 7.62 to 44.4 % of patients with diabetes, representing 0.9–5.3 million people [13–19].

Brazil, along with several other developing countries, does not have a national screening DR program, except regional initiatives of detection of cases that mainly rely on BIO [13–19].

Telemedicine has been established as a successful strategy for DR screening in various countries [6–8]. Digital retinographies can be obtained by non-medical trained personnel and sent to reading centers, where specialists are able to classify DR and categorize patients in terms of observation or referral.

The aim of this study was to compare binocular indirect ophthalmoscopy (BIO) and digital retinography (mydriatic and non-mydriatic) for the screening of DR in a large multicenter type 1 DM patients study.

## Methods

### Patients

This multicenter study included type 1 DM patients from 7 referral centers located in 4 different Brazilian regions (South, Southeast, Northeast and Center-West). Inclusion criterion was type 1 DM according to the American Diabetes Association [20] and exclusion criteria were any other eye diseases that produced retinal changes. Written informed consent was obtained from all patients, and the local ethics committee approved the study protocol.

### Methods

Each patient had both eyes examined, whenever possible, and underwent non-mydriatic retinography (NMR), mydriatic 2 field retinography (one field centered on the fovea and the other field centered on the optic disc) and mydriatic BIO.

At the reading center, mydriatic retinography centered on the fovea alone was considered as 1-field retinography (1FR) and combined mydriatic retinographies were considered as 2-field retinography (2FR).

Mydriasis was obtained with 1 % tropicamide drops. BIO was performed with an Eyetec Ophthalmoscope (Eyetec, São Carlos-SP, Brazil) and a 20 diopter lens (Volk Optical, Mentor, OH, USA) by an experienced retinal specialist in each center. After BIO, each eye was classified for diabetic retinopathy and maculopathy according to the American Academy of Ophthalmology guidelines [10] (Tables 1, 2).

**Table 1:** Diabetic retinopathy classification

Severity level	Ophthalmoscopy changes	Retinography changes	Outcome
Absent	No alterations	No alterations	Observation^a^
Mild non-proliferative	Microaneurysms only	At least one hemorrhage or microaneurysm	Observation^a^
Moderate non-proliferative	More than “microaneurysms only” and less than “Severe non- proliferative”	Four or more hemorrhages in only one hemi-field (superior and inferior hemi-fields separated by the line passing through the center of the macula and the optic disc)	Referral
Severe non-proliferative	Any of the following: retinal hemorrhages in each of the four quadrants; venous beading in 2 quadrants; intraretinal microvascular abnormalities (IRMA) in one quadrant *AND* absence of proliferative signs	Any of the following: Four or more hemorrhages in the superior AND inferior hemi-fields Venous beading IRMA	Referral
Proliferative	One or more of the following: neovascularization, vitreous or pre-retinal hemorrhage	Any of the following: Active neovessels Vitreous hemorrhage	Referral

^a^Except in cases of apparently present macular edema (see Table 2), in which the outcome was referral

**Table 2:** Diabetic maculopathy classification

Severity level	Ophthalmoscopy changes	Retinography changes	Outcome
Apparently absent diabetic macular edema	Absence of retinal thickening or hard exudates on the posterior pole	No alterations	Observation^a^
Apparently present diabetic macular edema	Retinal thickening or hard exudates on the posterior pole	Lesions within 2 disc diameters from the foveal center; any hard exudate	Referral

^a^Only in cases of absent or mild non-proliferative diabetic retinopathy: in more severe stages of DR, the outcome is referral

Clinical outcome was either observation or referral to the ophthalmologist. For the present study, the following criteria were adopted for referral: moderate or severe non-proliferative diabetic retinopathy, proliferative diabetic retinopathy, or apparently present diabetic macular edema [21, 22].

For some sub-analysis, eyes were separated in two groups: the least severe group (absent or mild to moderate non-proliferative diabetic retinopathy) and the most severe group (severe non-proliferative or proliferative diabetic retinopathy).

Retinographies were performed with a Canon CR-s Digital Retinal Camera (Canon Inc, Melville, NY, USA); images were obtained at 45° angles and 3168 × 4752 pixels resolution. Retinographies were taken by previously trained non-medical personnel and sent in the DICON (Digital Imaging and Communications in Medicine) protocol to the reading center (MediViewWeb software, version 4.0.75, Medilab, Rio de Janeiro, Brazil). All retinographies in each center were taken by the same trained individual.

**Fig. 1:**
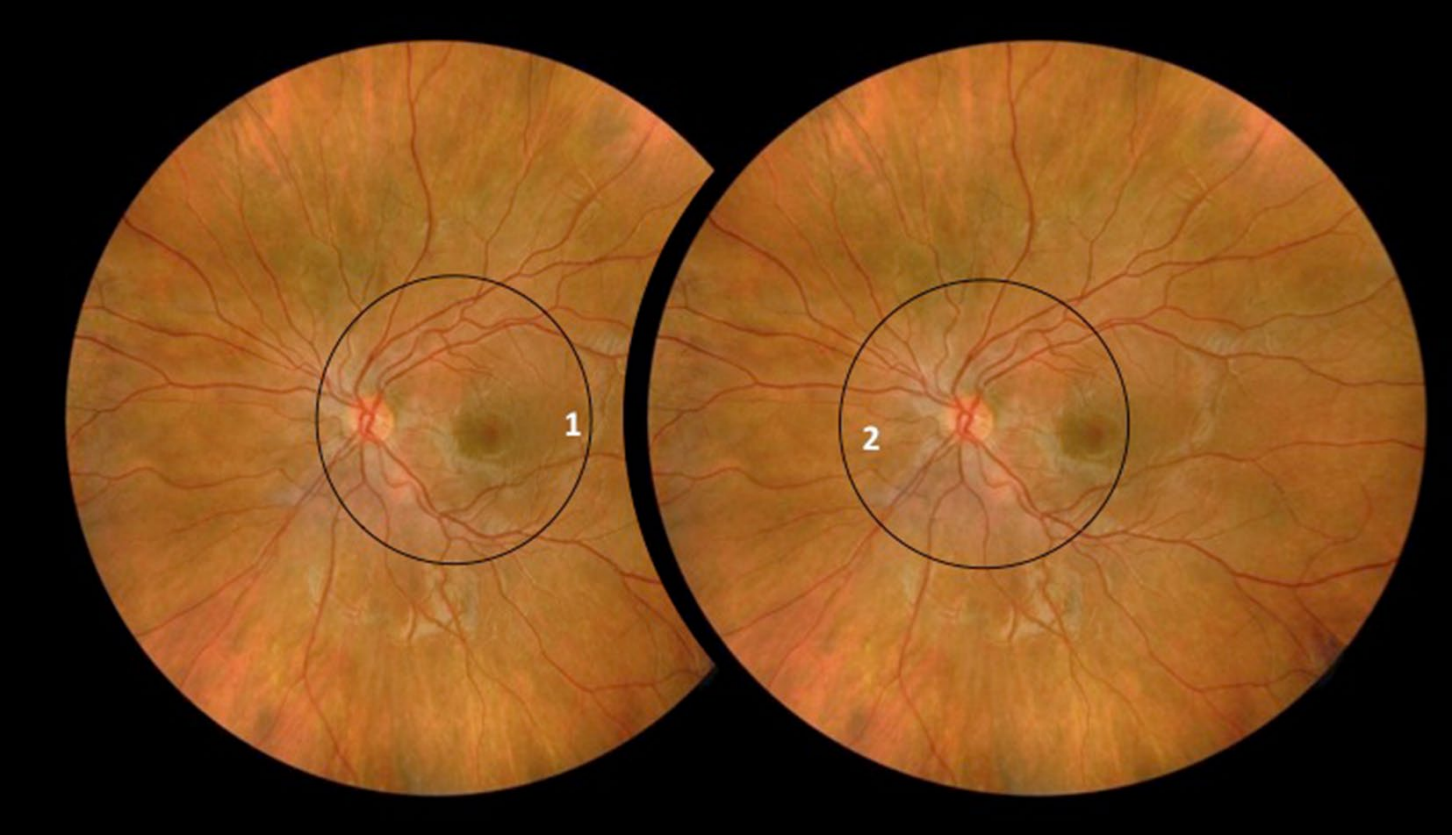
Retinography areas. *1* For fovea-centered retinographies, images should display both temporal vascular arcades and at least half disc diameter of retina nasal to the optic disc. *2* For optic disc-centered retinographies, images should display the four vascular arcades and at least one disc diameter of retina temporal to the fovea

Readings were performed by retinal specialists and began with the evaluation of the quality of images, regarding transparency of the media, focus and image boundaries. Third order retinal vessels needed to be identified. Images needed to display (Fig. 1):

- For fovea-centered retinographies: both temporal vascular arcades and at least half disc diameter of retina nasal to the optic disc;
- For optic disc-centered retinographies: the four vascular arcades and at least one disc diameter of retina temporal to the fovea.

The reading sequence of retinographies was as follows: first, NMR of each eye followed by 1FR and finally 2FR. Reading was performed with a 19″ monitor, and zoom was used according to the readers’ convenience. Reading was performed in an independent and masked fashion, and no additional clinical information was given to the readers.

The classification system for retinographies was adapted from the Scottish Diabetic Retinopathy screening program [22] (Tables 1, 2).

To allow comparison of a clinical diagnostic method (BIO) with digital retinographies, we proposed an equivalence classification system (Tables 1, 2).

Finally, for patients analyses instead of eyes analyses, we considered the eye with the most severe classification, and 2FR, preferably. All eyes with evidence of laser treatment were considered “proliferative diabetic retinopathy”.

The A1C was measured by HPLC (TOSOH G7, Luxembourg, Belgium) (nv: 4.0–5.6 %).

### Statistics

All data were collected in MS Excel 2010 files (Microsoft Corporation, Redmond, WA, USA). Statistical analyses were performed with “R” software, version 3.1.1 (R Foundation for Statistical Computing, Vienna, Austria) and SPSS 19.0 for Windows (SPSS Inc., Chicago, IL, USA).

For agreement analyses, we used Kappa statistics [23], with the following ranges of agreement: 0 to 0.19—poor agreement; 0.2 to 0.39—fair agreement; 0.4 to 0.59—moderate agreement; 0.6 to 0.79—substantial agreement and 0.8 to 1.00—almost perfect agreement [24]. Exact Fischer or Chi square tests were used for unpaired variables, and the McNemar test was used for paired variables. The 5 % level of significance was used. Binomial distribution was used for confidence interval for proportions calculation. The Mann–Whitney test was used to assess group differences for quantitative variables [25–27].

## Results

**Fig. 2:**
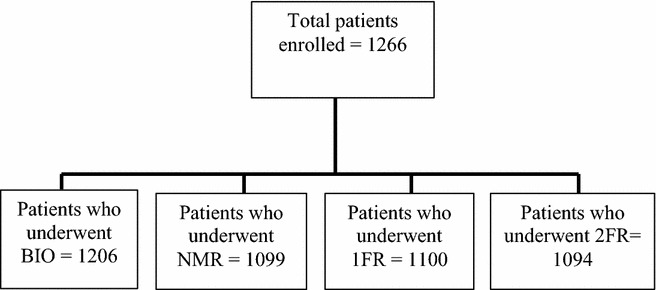
Number of patients in each diagnostic method. *BIO* binocular indirect ophthalmoscopy, *NMR* non-mydriatic retinography, *1 FR* 1 field mydriatic retinography, *2 FR* 2 field mydriatic retinography

The number of participants who underwent at least two methods was 1266, with 56.8 % women. Comparison of the clinical outcome (observation or referral) between the group of patients who underwent all methods and the group of patients that missed at least one method showed homogeneity (p = 0.2471). The number of patients that underwent each method is displayed in Fig. 2. The number of participants who had both eyes evaluated by the four methods (BIO, NMR, 1FR, 2FR) was 1023.

The following data are given as mean (standard deviation) and median (range). Age (years), diabetes duration (years) and hemoglobin A1C (percentage) were respectively: 31 (12) and 29 (8–73); 16 (9) and 15 (0–56); and 10.6 (2.8) and 10.4 (5.0–18.6).

**Fig. 3:**
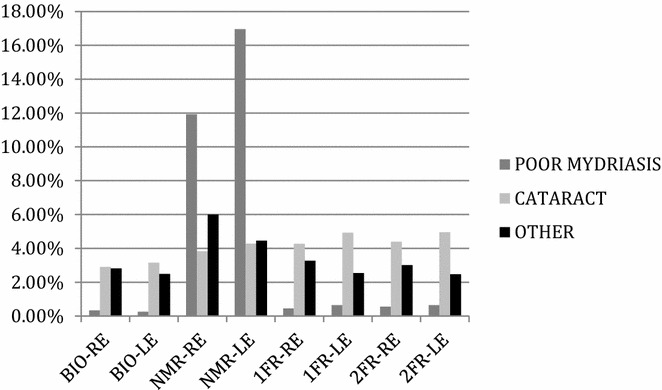
Causes for poor quality of images. *BIO* binocular indirect ophthalmoscopy, *NMR* non-mydriatic retinography, *1 FR* 1 field mydriatic retinography, *2 FR* 2 field mydriatic retinography, *RE* right eye, *LE* left eye

Quality was considered adequate in over 93, 74, 91 and 91 % of eyes for BIO, NMR, 1FR and 2FR, respectively. The main causes for poor quality are shown in Fig. 3.

DR and maculopathy classification in each center, in the four different Brazilian regions, is shown in Table 3. Classifications are based on the most severely affected eye and 2FR strategy, preferably.

**Table 3:** Diabetic retinopathy and maculopathy classification in each center, in four different Brazilian regions (Southeast, South, Northeast and Center-West)

	Center 1 Southeast	Center 2 Southeast	Center 3 Southeast	Center 4 Southeast	Center 5 South	Center 6 Northeast	Center 7 Center-West	Total
DR-absent	109 (60.6 %)	118 (45.9 %)	71 (41.0 %)	31 (32.6 %)	89 (49.4 %)	82 (50.3 %)	108 (49.5 %)	608 (48.0 %)
DR-NP mild	35 (19.4 %)	91 (35.4 %)	53 (30.6 %)	33 (34.7 %)	44 (24.4 %)	42 (25.8 %)	54 (24.8 %)	352 (27.8 %)
DR-NP moderate	10 (5.6 %)	12 (4.7 %)	5 (2.9 %)	6 (6.3 %)	8 (4.4 %)	4 (2.5 %)	7 (3.2 %)	52 (4.1 %)
DR-NP severe	8 (4.4 %)	9 (3.5 %)	4 (2.3 %)	0 (0.0 %)	3 (1.7 %)	4 (2.5 %)	6 (2.8 %)	34 (2.7 %)
DR-proliferative	14 (7.8 %)	20 (7.8 %)	29 (16.8 %)	18 (19.0 %)	27 (15.0 %)	22 (13.5 %)	29 (13.3 %)	159 (12.6 %)
DR-impossible	4 (2.2 %)	7 (2.7 %)	11 (6.4 %)	7 (7.4 %)	9 (5.0 %)	9 (5.5 %)	14 (6.4 %)	61 (4.8 %)
Total retinopathy	180 (100 %)	257 (100 %)	173 (100 %)	95 (100 %)	180 (100 %)	163 (100 %)	218 (100 %)	1266 (100 %)
MAC-apparently absent	141 (78.3 %)	201 (78.2 %)	137 (79.2 %)	72 (75.8 %)	144 (80.0 %)	124 (76.1 %)	161 (73.9 %)	980 (77.4 %)
MAC-apparenlty present	32 (17.8 %)	42 (16.4 %)	14 (8.1 %)	13 (13.7 %)	18 (10.0 %)	25 (15.3 %)	35 (16.1 %)	179 (14.1 %)
MAC-impossible	7 (3.9 %)	14 (5.4 %)	22 (12.7 %)	10 (10.5 %)	18 (10.0 %)	14 (8.6 %)	22 (10.1 %)	107 (8.5 %)
Total maculopathy	180 (100 %)	257 (100 %)	173 (100 %)	95 (100 %)	180 (100 %)	163 (100 %)	218 (100 %)	1266 (100 %)

*DR* diabetic retinopathy, *NP* non-proliferative, *MAC* maculopathy, *BIO* binocular indirect ophthalmoscopy

Considering DR and maculopathy classification in the worst eye, 69 % of patients were “observable” and 31 % “referable” (see “Methods”).

**Fig. 4:**
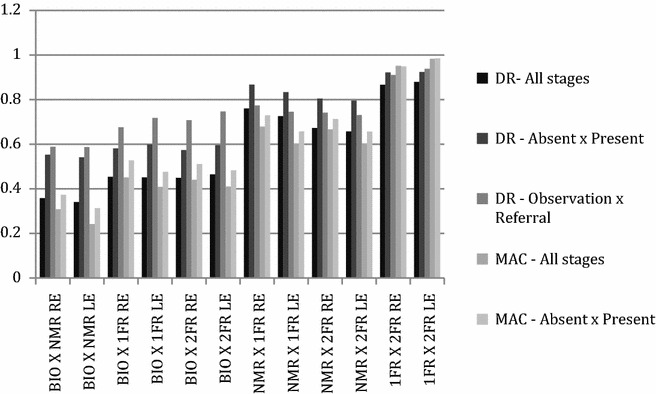
Kappa agreement between methods. *DR* diabetic retinopathy, *MAC* diabetic maculopathy, *BIO* binocular indirect ophthalmoscopy, *NMR* non-mydriatic retinography, *1 FR* 1 field mydriatic retinography, *2 FR* 2 field mydriatic retinography, *RE* right eye, *LE* left eye

When BIO and mydriatic retinography screening methods were compared regarding DR classification for the main outcome (observation versus referral), agreement was substantial (kappa 0.67–0.74). However, when BIO and non-mydriatic screening methods were compared regarding DR classification for the same outcome, agreement was only moderate (kappa 0.58). When BIO and the retinographies strategies were compared regarding maculopathy classification, agreement was fair to moderate (kappa 0.24–0.45). Agreement analysis is shown in Fig. 4.

Poor mydriasis was the main cause of poor quality in NMR, occurring in 11.92 % of right eyes and 16.96 % of left eyes. Poor mydriasis accounted for less than 1 % of poor quality in the mydriatic methods.

Poor mydriasis did not allow classification in 8.5 % of right eyes that underwent NMR, ranging 3.1–10.6 % in the various centers, and 12.1 % of left eyes, ranging 7.1–20 % in the various centers.

Poor mydriasis usually led readers to overestimate the DR stage in NMR, when compared to the classification for the same eyes with mydriatic methods. About 70 % of eyes with poor mydriasis in NMR that received a different classification in 1FR or 2FR were considered more severely affected in NMR (67.9 and 73.3 % of right eyes and 68.3 and 78.7 % of left eyes with 1FR for retinopathy and maculopathy, respectively; 67.7 and 73.1 % of right eyes and 68.1 and 78.0 % of left eyes with 2FR for retinopathy and maculopathy, respectively).

Patient age and retinopathy severity were associated with poor mydriasis or impossibility of classification in NMR. The average age of the patients with good mydriasis in NMR was 28 years old, whereas 36 years old for patients with poor mydriasis in NMR (p < 0.0001). The worse the severity of diabetic retinopathy, according to 2FR classification, the higher the percentage of eyes with poor mydriasis in NMR. For this sub-analysis, eyes were separated into two groups: the least severely affected and the most severely affected (see “Methods”). Eyes with poor mydriasis in NMR in the “least severe” group were 12 % right eyes and 15.1 % left eyes, whereas eyes with poor mydriasis in NMR in the “most severe” group were 62 % right eyes and 56 % left eyes. All differences were statistically significant (p < 0.0001).

BIO allowed for the classification of most eyes (58.1 %) that could not be properly classified by 2FR strategy.

## Discussion

We found a substantial agreement (kappa 0.67–0.74) between BIO and mydriatic retinographies for diabetic retinopathy classification regarding clinical outcome (observation vs referral), but only moderate agreement (kappa 0.58) between BIO and non-mydriatic retinography. The main factor that prevented diabetic retinopathy classification in the present study was poor mydriasis in NMR.

Our results agree with previous reports [28–30] that also observed problems with NMR. Left eyes were most affected by poor mydriasis (Fig. 3): after the first retinography taken on the right eye, both pupils contract because of the flash, making it more difficult to obtain enough mydriasis on the second (left) eye. Poor mydriasis in NMR promoted classification errors, because of shadows or reflexes on the image, usually leading the reader to overestimate the severity in about 70 % of eyes. Poor mydriasis was observed more often in older patients or in eyes with more severe stages of retinopathy. Poor mydriasis was the only modifiable factor that prevented a readable image, since it dropped considerably in the mydriatic methods, whereas all other factors persisted (Fig. 3). Our findings advise against a diabetic retinopathy screening strategy with conventional non-mydriatic retinography. Ultra-wide field retinography is not currently a possible screening strategy in developing countries because of high costs [31–33].

Mydriatic 1-field and 2-field retinography strategies had an almost perfect agreement (kappa 0.91–0.93). However, a small percentage (5.08 %) of observable patients according to 1FR were considered referable according to 2FR. These patients probably had significant changes in the nasal retina. As it is extremely easy and not time-consuming to obtain the second photo once the pupils are dilated, we believe that our findings support a 2-field protocol screening strategy [29].

Our results showed that BIO agreed substantially with digital retinography only regarding the clinical outcome (observation or referral), but they did not show a good agreement in DR classification if all stages were considered. BIO and retinographies did not show a good agreement regarding diabetic maculopathy (Fig. 4), either. BIO usually does not allow for identification of minor changes such as microaneurysms, retinal neovessels or mild macular edema [34], and probably that was the reason why BIO did not show a good agreement with retinographies regarding retinopathy and maculopathy classification. According to BIO, only 4 % of eyes had maculopathy, whereas maculopathy was present in 9 % of eyes according to 2FR. Additionally, BIO can only be performed by a specialist, precluding a telemedicine-based strategy.

The ETDRS protocol is still the gold standard for diabetic retinopathy classification, but it is very time-consuming and requires a highly trained professional for image acquisition [35]. Protocols with a smaller number of fields [6, 36, 37], as well as protocols with digital retinographies [38, 39], have already been validated for DR screening. In the present study, we used a 2-field digital retinography protocol that required less than 5 min per patient.

Besides poor mydriasis, some documentation for the present study was lacking because of improper technique. We believe this problem may be resolved with better training of the personnel, since there is a learning curve for retinography acquisition [40]. Some anatomical features of the eye may also render the examination or the reading especially difficult, for example, high myopic eyes with a hypopigmented fundus [41].

The referral criteria for the present study encompassed patients with moderate non-proliferative diabetic retinopathy or worse [21]. Patients with moderate non-proliferative retinopathy are clinically followed and not treated, and the inclusion of this stage might have overestimated the referral burden. However, our study still concluded that such classification would decrease the referral burden by about 70 %.

ETDRS classified diabetic macular edema into clinically significant (treatable) and non-clinically significant (observable). Our study evaluated only indirect signs of macular edema, such as hard exudates, but not retinal thickening itself. Hence, instead of using ETDRS classification, we employed the classification “apparently present” or “apparently absent” [10], with the former corresponding to both “observable maculopathy” and “referable maculopathy” categories from the Scottish grading system [10]. All patients with an “apparently present” macular edema were considered referable in the present study.

The global increase in the prevalence of diabetes mellitus calls for the optimization of human and economic resources. One of the main advantages of telemedicine is that it allows for a decreased proportion of patients that otherwise would have been referred to a specialist [5, 7–9, 42]. Our results showed that around 70 % of examined patients would be observable, thereby alleviating the demand for the specialist.

BIO and retinography are both valid strategies for DR screening. The main advantages of BIO are its ease of handling and low cost [13]. BIO may perform superiorly in certain circumstances in comparison to retinographies, as in cases of media opacities or poor patient collaboration, for example. In our study, BIO allowed for the classification of most eyes that could not have been properly classified by 2FR strategy. BIO has some disadvantages, such as not being sensitive enough to detect minor signs of DR [34]. The main disadvantages of the retinography strategy are its cost and its poor performance in unfavorable anatomical features, as stated above. However, the central issue regarding the comparison of BIO and retinographies is that the former depends on the presence of a specialist, whereas the latter is compatible with telemedicine strategies. The current and future demands for retinal evaluation due to the global diabetes epidemics is not compatible with the presence of a specialist for every fundus exam [43]; telemedicine also is a feasible screening strategy for remote locations or areas with an uneven distribution of specialists.

This is the first Brazilian multicenter study that compared several DR screening methods in type 1 DM patients; we believe that our results could be extrapolated to other developing countries with remote areas and an uneven distribution of specialists.

This study had limitations. First, it was not designed to compare DR strategies to the gold-standard, ETDRS 7-field protocol. However, the ETDRS protocol is not suitable as a screening technique since it requires expert photographers and is time-consuming [44]. Additionally, the present study included only patients enrolled in referral urban centers. Further studies should also enroll patients from other settings, such as rural areas or primary care units. Future research on telemedicine and DR screening should also evaluate the practical impact of such measures in the decline of diabetic retinopathy as a major cause of blindness, as was the case in England and Wales [44]. Automatic detection of DR combined with telemedicine is also a promising field of research [44], as well as the study of other layers of interventions, such as telemedicine with health education and promotion, in order to improve DR screening coverage [45].

## Conclusions

Our study showed that BIO and a telemedicine-based mydriatic retinography protocol are equivalent methods for DR screening. Because of its cost-effectiveness and its role in regions where specialists are unavailable, we recommend the telemedicine-based mydriatic strategy for DR screening.

## Abbreviations

BIO: binocular indirect ophthalmoscopy
DR: diabetic retinopathy
DM: diabetes mellitus
ETDRS: the early treatment diabetic retinopathy study
NMR: non-mydriatic retinography
1FR: 1-field retinography
2FR: 2-field retinography
DICON: digital imaging and communications in medicine
NP: non-proliferative
MAC: maculopathy
RE: right eye
LE: left eye

## References

[1] Zheng Y, He M, Congdon N (2012). The worldwide epidemic of diabetic retinopathy. Indian J Ophthalmol.

[2] Yau JW, Rogers SL, Kawasaki R, Lamoureux EL, Kowalski JW, Bek T (2012). Meta-Analysis for Eye Disease (META-EYE) Study Group. Global prevalence and major risk factors of diabetic retinopathy. Diabetes Care.

[3] Li R, Zhang P, Barker LE, Chowdhury FM, Zhang X (2010). Cost-effectiveness of interventions to prevent and control diabetes mellitus: a systematic review. Diabetes Care.

[4] Au A, Gupta O (2011). The economics of telemedicine for vitreoretinal diseases. Curr Opin Ophthalmol.

[5] Stefánsson E (2006). Prevention of diabetic blindness. Br J Ophthalmol.

[6] Looker HC, Nyangoma SO, Cromie DT, Olson JA, Leese GP, Black MW (2014). On behalf of the Scottish Diabetes Research Network Epidemiology Group and the Scottish Diabetic Retinopathy Collaborative. Rates of referable eye disease in the Scottish National Diabetic Retinopathy Screening Programme. Br J Ophthalmol.

[7] Forster AS, Forbes A, Dodhia H, Connor C, Du Chemin A, Sivaprasad S (2013). Changes in detection of retinopathy in type 2 diabetes in the first 4 years of a population-based diabetic eye screening program. Diabetes Care.

[8] Hautala N, Aikkila R, Korpelainen J, Keskitalo A, Kurikka A, Falck A (2014). Marked reductions in visual impairment due to diabetic retinopathy achieved by efficient screening and timely treatment. Acta Ophthalmol.

[9] Agardh E, Agardh CD, Hansson-Lundblad C (1993). The five-year incidence of blindness after introducing a screening programme for early detection of treatable diabetic retinopathy. Diabet Med.

[10] Wilkinson CP, Ferris FL, Klein RE, Lee PP, Agardh CD, Davis M (2003). Representing the Global Diabetic Retinopathy Project Group. Proposed international clinical diabetic retinopathy and diabetic macular edema disease severity scales. Ophthalmology..

[11] Photocoagulation for diabetic macular edema (1985). Early treatment diabetic retinopathy study report number 1. Early treatment diabetic retinopathy Study research group. Arch Ophthalmol.

[12] IDF Diabetes Atlas. Available from http://www.idf.org/diabetesatlas. Accessed 1 July 2015.

[13] Schellini SA, Carvalho GM, Rendeiro FS, Padovani CR, Hirai FE (2014). Prevalence of diabetes and diabetic retinopathy in a Brazilian population. Ophthalmic Epidemiol.

[14] Jost BS, Hilgemberg E, Rodrigues EB, Daniotti AF, Bonamigo EL (2010). Prevalence of diabetic retinopathy in patients affected by type 2 diabetes mellitus in the city of Luzerna—SC. Arq Bras Oftalmol..

[15] Escarião PHG, Arantes TEF, Figueiroa Filho NC, Urtiga RD, Florêncio TLT, Arcoverde ALAL (2008). Epidemiology and regional differences of diabetic retinopathy in Pernambuco. Brazil. Arq Bras Oftalmol..

[16] Guedes MF, Portes AJF, Couto Junior AS, Nunes JS, Oliveira RCC (2009). Prevalence of the diabetic retinopathy in a Family’s Health Program unity. Rev Bras Oftalmol..

[17] Souza EV, Souza NV, Rodrigues MLV (2004). Diabetic retinopathy among patients assisted by a multidisciplinary program at the University Hospital of Ribeirão Preto, São Paulo—USP. Arq Bras Oftalmol.

[18] Esteves JF, Kramer CK, Azevedo MJ, Stolz AP, Roggia MF, Larangeira A (2009). Prevalence of diabetic retinopathy in patients with type 1 diabetes mellitus. Rev Assoc Med Bras.

[19] Ramos SR, Sabbag FP, Busato D, Miranda AB, Moreira Júnior CA (1999). Diabetic retinopathy: study from a Diabetic Association. Arq Bras Oftal..

[20] American Diabetes Association (2014). Standards of medical care 2014. Diabetes Care.

[21] Olson JA, Strachan FM, Hipwell JH, Goatman KA, McHardy KC, Forrester JV (2003). A comparative evaluation of digital imaging, retinal photography and optometrist examination in screening for diabetic retinopathy. Diabet Med..

[22] Philip S, Fleming AD, Goatman KA, Fonseca S, McNamee P, Scotland GS (2007). The efficacy of automated ‘‘disease/no disease’’ grading for diabetic retinopathy in a systematic screening programme. Br J Ophthalmol.

[23] Kraemer HC, Periyakoil VS, Noda A (2002). Tutorial in biostatistics. Kappa coefficients in medical research. Statist Med..

[24] Landis JR, Koch GG (1977). The measurement of observer agreement for categorical data. Biometrics..

[25] Siegel S, Castellan NJ (1988). Nonparametric statistics.

[26] Pereira JCR (2010). Bioestatística em outras palavras.

[27] Fleiss JL, Levin B, Paik MC (2003). Statistical methods for rates and proportions.

[28] Murgatroyd H, Ellingford A, Cox A, Binnie M, Ellis JD, MacEwen CJ (2004). Effect of mydriasis and different field strategies on digital image screening of diabetic eye disease. Br J Ophthalmol.

[29] Herbert HM, Jordan K, Flanagan DW (2003). Is screening with digital imaging using one retinal view adequate?. Eye..

[30] Shi L, Wu H, Dong J, Jiang K, Lu X, Shi J (2015). Telemedicine for detecting diabetic retinopathy: a systematic review and meta-analysis. Br J Ophthalmol.

[31] Soliman AZ, Silva PS, Aiello LP, Sun JK (2012). Ultra-wide field retinal imaging in detection, classification, and management of diabetic retinopathy. Semin Ophthalmol..

[32] Witmer MT, Kiss S (2013). Wide-field imaging of the retina. Surv Ophthalmol.

[33] Silva PS, Cavallerano JD, Tolson AM, Rodriguez J, Rodriguez S, Ajlan R (2015). Real-time ultrawide field image evaluation of retinopathy in a diabetes telemedicine program. Diabetes Care.

[34] Kinyoun JL, Martin DC, Fujimoto WY, Leonetti DL (1992). Ophthalmoscopy versus fundus photographs for detecting and grading diabetic retinopathy. Invest Ophthalmol Vis Sci.

[35] Kernt M, Hadi I, Pinter F, Seidensticker F, Hirneiss C, Haritoglou C (2012). Assessment of diabetic retinopathy using nonmydriatic ultra-widefield scanning laser ophthalmoscopy (Optomap) compared with ETDRS 7-field stereo photography. Diabetes Care.

[36] Liesenfeld B, Kohner E, Piehlmeier W, Kluthe S, Aldington S, Porta M (2000). A telemedical approach to the screening of diabetic retinopathy: digital fundus photography. Diabetes Care.

[37] Tran THC, Rahmoun J, Hui Bon Hoa AA (2009). Screening for diabetic retinopathy using a three-field digital nonmydriatic fundus camera in the North of France. J Français d’ophthalmologie..

[38] Li HK, Danis RP, Hubbard LD, Florez-Arango JF, Esquivel A, Krupinski EA (2011). Comparability of digital photography with the ETDRS film protocol for evaluation of diabetic retinopathy severity. Invest Ophthalmol Vis Sci.

[39] Lin DY, Blumenkranz MS, Brothers RJ, Grosvenor DM (2002). The sensitivity and specificity of single-field nonmydriatic monochromatic digital fundus photography with remote image interpretation for diabetic retinopathy screening: a comparison with ophthalmoscopy and standardized mydriatic color photography. Am J Ophthalmol.

[40] Ogunyemi O, Moran E, Patty Daskivich L, George S, Teklehaimanot S, Ilapakurthi R (2013). Autonomy versus automation: perceptions of nonmydriatic camera choice for teleretinal screening in an urban safety net clinic. Telemed J E Health..

[41] Jonas JB, Xu L (2014). Histological changes of high axial myopia. Eye..

[42] Looker HC, Nyangoma SO, Cromie D, Olson JA, Leese GP, Black M (2012). On behalf of the Scottish Diabetic Retinopathy Screening Collaborative and the Scottish Diabetes Research Network Epidemiology Group. Diabetic retinopathy at diagnosis of type 2 diabetes in Scotland. Diabetologia..

[43] Silva PS, Aiello LP (2015). Telemedicine and eye examinations for diabetic retinopathy. A time to maximize real-world outcomes. JAMA Ophthalmol..

[44] Zimmer-Galler IE, Kimura AE, Gupta S (2015). Diabetic retinopathy screening and the use of telemedicine. Curr Opin Ophthalmol.

[45] Mansberger SL, Sheppler C, Barker G, Gardiner SK, Demirel S, Wooten K (2015). Long-term comparative effectiveness of telemedicine in providing diabetic retinopathy screening examinations: a randomized clinical trial. JAMA Ophthalmol..

